# Cardiovascular Disease Risk Factor Profiles of 263,356 Older Australians According to Region of Birth and Acculturation, with a Focus on Migrants Born in Asia

**DOI:** 10.1371/journal.pone.0115627

**Published:** 2015-02-19

**Authors:** Shuyu Guo, Robyn M. Lucas, Grace Joshy, Emily Banks

**Affiliations:** 1 National Centre for Epidemiology and Population Health, Research School of Population Health, The Australian National University, Canberra, Australia; 2 The Sax Institute, Sydney, New South Wales, Australia; 3 Telethon Kids Institute, University of Western Australia, Perth, Australia; University of Verona, Ospedale Civile Maggiore, ITALY

## Abstract

Risk factors for cardiovascular disease (CVD), such as obesity, diabetes, hypertension and physical inactivity, are common in Australia, but the prevalence varies according to cultural background. We examined the relationship between region of birth, measures of acculturation, and CVD risk profiles in immigrant, compared to Australian-born, older Australians. Cross-sectional data from 263,356 participants aged 45 and over joining the population-based 45 and Up Study cohort from 2006–2008 were used. Prevalence ratios for CVD risk factors in Australian- versus overseas-born participants were calculated using modified Poisson regression, adjusting for age, sex and socioeconomic factors and focusing on Asian migrants. The association between time resident in Australia and age at migration and CVD risk factors in Asian migrants was also examined. Migrants from Northeast (n = 3,213) and Southeast Asia (n = 3,942) had lower levels of overweight/obesity, physical activity and female smoking than Australian-born participants (n = 199,356), although differences in prevalence of overweight/obesity were sensitive to body-mass-index cut-offs used. Compared to Australian-born participants, migrants from Northeast Asia were 20–30% less likely, and from Southeast Asia 10–20% more likely, to report being treated for hypertension and/or hypercholesterolaemia; Southeast Asian migrants were 40–60% more likely to report diabetes. Northeast Asian-born individuals were less likely than Australian-born to have 3 or more CVD risk factors. Diabetes, treated hypertension and hypercholesterolaemia occurred at relatively low average body-mass-index in Southeast Asian migrants. The CVD risk factor profiles of migrants tended to approximate those of Australian-born with increasing acculturation, in both favourable (e.g., increased physical activity) and unfavourable directions (e.g., increased female smoking). Minimizing CVD risk in migrant populations may be achieved through efforts to retain the healthy facets of the traditional lifestyle, such as a normal body mass index and low prevalence of smoking in women, in addition to adopting healthy aspects of the host country lifestyle, such as increased physical activity.

## Introduction

In recent years, advances in clinical and public health interventions have resulted in significant progress in reducing cardiovascular disease (CVD) related morbidity and mortality in many developed countries[1]. However, CVDs remain the leading cause of morbidity and mortality worldwide, accounting for 30% of all deaths[2]. There is also troubling evidence that the prevalence of risk factors for CVD, such as overweight/obesity, diabetes, hypertension and physical inactivity is increasing[2]. While these risk factors are individually important, there is a steady increase in risk of all-cause and cardiovascular disease mortality as the number of risk factors present increases[3,4].

International migration from non-western to western countries is increasing[5]. Migrants from diverse cultural backgrounds have health characteristics that are different from the populations of their host countries[6]. Evidence suggests that the prevalence of CVD risk factors may vary considerably among heterogeneous migrant groups according to birth place[6–8], and also differ according to degree of acculturation, measured by such factors as duration of residence in the host country, age at migration and proficiency in the host language[9,10].

Australia is among one of the most multicultural societies in the world, with 27% of the population born overseas. Asian migrants were one of the most rapidly growing population groups in the latter part of the 20^th^ century[11]. Understanding the CVD risk profile and how this changes as migrants become acculturated to the host country is important to provide an evidence-base to guide programs to improve health outcomes in migrants.

Recent studies of migrants to Australia have examined changes in only a single risk factor [12–15], with information on multiple CVD risk factors available only within studies that are now over 10 years old [6,16–18]. Furthermore, CVD risk profiles of recently arrived migrant groups from Asia have not been explored in detail. Thus research to date may not accurately reflect the current CVD risk situation among overseas-born populations in Australia.

We used cross-sectional data from the 45 and Up Study to compare multiple CVD risk factors in Australian-born and overseas-born participants according to region of birth, with an emphasis on Asian migrants, and examined the association between acculturation variables and these risk factors in Asian migrants.

## Methods

### Study Population

The 45 and Up Study is a large population-based cohort study of people aged 45 years and over, living in New South Wales, Australia [19]. This age group has a high prevalence of CVD risk factors and related morbidity and mortality. A total of 266,097 individuals, recruited following random sampling from the Medicare Australia database, completed a self-administrated baseline questionnaire and informed consent distributed between January 2006 and December 2008. Baseline questionnaire data included information on socio-demographic and lifestyle factors, self-reported medical history, height and body weight, and physical activity. Participants were also asked about their country of birth, and, for those born outside of Australia, they were also asked to provide the year they moved to Australia.

The data for the project were obtained from a third party, namely the Sax Institute, which is the data custodian for the 45 and Up Study. Data are available through application to the Sax Institute (details are available at https://www.saxinstitute.org.au/our-work/45-up-study/) or through contacting 45andUp.research@saxinstitute.org.au.

### Region of birth

Based on the question “In which country were you born?”, “overseas-born” was defined as being born in a place other than Australia. Countries of birth were categorized into five major groups according to a modified version of the Standard Australian Classification of Countries (SACC, 2011)[20]: Australia, Europe, Northeast Asia (i.e. China, Hong Kong, Taiwan, South Korea and Japan), Southeast Asia (i.e. Burma, Cambodia, Thailand, Vietnam, Indonesia, Malaysia, Philippines and Singapore) and Other.

### Socio-demographic variables

Five age categories were created: 45–49, 50–59, 60–69, 70–79, 80+. Marital status was categorized as “Married/living with a partner”, “Single”, and “Other (divorced, widowed or separated)”. Education was categorized as: “None”, “Intermediate/ high school/ trade/certificate diploma”, “University or higher”. Location of residence was defined as: “Major cities”, “Regional”, “Remote or very remote”. Household annual pre-tax income was categorized as <$20,000, $20,000-$39, 999, $40,000-$69,999, ≥$70,000. Health insurance was dichotomized as “has private health insurance” and “no private health insurance”.

### Acculturation variables

Two variables related to acculturation were examined: duration of residence in Australia and age migrated to Australia. Duration of residence in Australia was categorized as: 0–10, 11–20, 21–30, >30 years (with 0–10 as the reference, “less acculturated” group); age migrated to Australia was categorized as 0–10, 11–20, 21–30, >30 years, (with >30 as the reference group).

### Previous CVD incidents and CVD risk factors

Previous CVD was identified as self-reported heart disease, stroke or thrombosis according to the response to the question “Has a doctor ever told you that you have (following conditions)?” (yes/no). We examined 6 additional major CVD risk factors:

*Current Smoking*: Smoking status was categorized as current smoker, past smoker or never smoked based on the questions “Have you ever been a regular smoker?” “Are you a regular smoker now?” For multivariate analysis, current smoking and past smoking were used as two different risk factors, with never-smokers as the reference group.

*Diabetes*, *Current Treatment for Hypertension & Hypercholesterolaemia*: Data on diabetes were based on the self-report response to the question “Has a doctor ever told you that you have diabetes?” (yes/no). For female participants, if the age of giving birth to the last child was older than the age they were diagnosed with diabetes and they were not currently being treated for diabetes, they were categorized as “no diabetes”, to exclude gestational diabetes in this risk category. Current treatment for hypertension and current treatment for hypercholesterolaemia were recorded as being present if participants reported that they had been treated for these conditions in the previous month.

*Overweight/Obesity*: Body mass index (BMI) was calculated from self-reported height and weight. Obesity/overweight was defined as BMI≥25kg/m^2^ and used as a dichotomous variable (overweight/obesity vs. normal weight). We also conducted sensitivity analyses using the categorisation recommended by the World Health Organization for participants born in Asia [21], i.e. overweight/obesity defined as BMI ≥23kg/cm^2^.

*Physical Inactivity*: Respondents were asked “How many times did you do each of these activities last week? Walking continuously for at least 10 minutes/ Vigorous physical activity/ Moderate physical activity” “If you add up all the time you spent doing each activity last week, how much time did you spent altogether doing each type of activity? Walking continuously for at least 10 minutes/ Vigorous physical activity/ Moderate physical activity”. Physical activity level was dichotomized as physically inactive versus physically active (<150 minutes vs. ≥150 minutes of at least moderate-intensity physical activity, as recommended by the National Physical Activity Guidelines for Australians [22]).

*CVD risk profiles*: In addition to examining individual risk factors, we examined the combination of 6 major risk factors: current smoking, diabetes, current treatment for hypertension, current treatment for hypercholesterolaemia, BMI≥25 kg/m^2^ and physical inactivity (i.e. presence of 0, any 1 only, any 2 only and ≥3 risk factors).

### Statistical analysis

Of the 266,097 participants in the 45 and Up Study dataset used, 2741 (1%) were excluded from all analyses, because of missing data on country of birth. Missing data on CVD risk factors (BMI, n = 20356; physical activity, n = 13619; smoking status, n = 847) were categorized as “missing”, and included in the descriptive statistical analysis, but excluded from multivariate analysis that used the specific risk factor as the outcome variable.

The prevalence of previous CVD, individual CVD risk factor and CVD risk profiles were calculated by region of birth and for men and women separately. Given the high prevalence of overweight, physical inactivity and metabolic disorders in both Australian-born participants and migrants, odds ratios do not accurately reflect relative risks. Since logistic regression is thus not suitable for these analyses, modified Poisson regression models with a robust error variance[23] were used to estimate the association of region of birth and individual CVD risk factor and CVD risk profiles, using Australian-born participants as the reference group. Each model was stratified by sex. Prevalence ratios (PR) with 95% confidence intervals (CI) were estimated.

The two acculturation variables (i.e. duration of residence in Australia, age of migration) were used as categorical variables to examine the association with CVD risk factors, for Asian-born participants, stratified by sex and region of birth and adjusted for age and socio-demographic variables. Tests for linear trend for the acculturation variables of interest were performed by assigning the median value of each category to participants in that group and modelling as a continuous variable. Cumulative residual plots were used to investigate the linear functional form of continuous variables in the models. Missing values for covariates were included in the models as separate categories.

Previous CVD itself is a major risk factor for CVD morbidity and mortality in future. Patients who have had a heart attack, coronary angioplasty or other heart or blood vessel diseases may also substantially change their life styles. Thus, we performed additional sensitivity analysis to separately examine the results in participants with and without self-reported previous CVD.

All analyses were conducted using SAS 9.3 [24].

The conduct of the 45 and Up Study was approved by the University of New South Wales Human Research Ethics Committee (HREC). Ethical approval for this sub-study was provided by the NSW Population and Health Services Research Ethics Committee and the Human Research Ethics Committee of the Australian National University.

## Results

### Sample characteristics

A total of 263,356 participants were included in the analyses; 25% were overseas-born immigrants (3,213 from Northeast Asia, 3,942 from Southeast Asia, 41,061 from Europe, 15,963 from other regions). Table 1 shows the socio-demographic characteristics of these participants by region of birth (data for migrants from other regions are not presented).

**Table 1:** Socio-demographic characteristics of participants in the 45 and Up Study whose data were included in this analysis, by region of birth.

Country of Birth	Australia	Northeast Asia	Southeast Asia	Europe
	(n = 199356)	(n = 3213)	(n = 3942)	(n = 41061)
	%(n)	%(n)	%(n)	%(n)
**Sex**				
Female	55 (108593)	55 (1760)	57 (2347)	49 (20222)
Male	45 (90403)	45 (1453)	43 (1595)	51 (20839)
**Age**				
Mean ± SD	62 ± 11.08	59 ± 10.78	59 ± 10.78	65 ± 11.32
45–49	12 (23661)	18 (563)	16 (650)	8 (3153)
50–59	34 (66995)	43 (1393)	47 (1835)	26 (10561)
60–69	28 (55324)	18 (567)	22 (849)	32 (13327)
70–79	16 (32535)	13 (418)	8 (318)	19 (7735)
80+	10 (20087)	8 (272)	7 (290)	15 (6285)
**Education**				
None	12 (23178)	8 (243)	11 (428)	14 (5724)
Intermediate/High school/Trade/certificate/diploma	66 (13026)	48 (1544)	47 (1841)	61 (25365)
University or higher	22 (43110)	42 (1355)	40 (1592)	22 (9084)
**Annual household pre-tax income**				
<$20,000	19 (37548)	25 (824)	25 (973)	22 (9226)
$20,000-$39,999	18 (35484)	15 (466)	15 (588)	18 (7287)
$40,000-$69,999	18 (36157)	17 (541)	18 (713)	16 (6458)
≥$70,000	24 (47728)	18 (576)	18 (740)	21 (8553)
**Marital status**				
Current married/live with partner	74 (148116)	80 (2577)	76 (2994)	73 (30100)
Other	19 (37179)	14 (457)	17 (676)	21 (8589)
Never married	6 (12920)	5 (169)	6 (251)	5 (2123)
**Location of residence**				
Major cities	40 (79514)	91 (2939)	83 (3280)	54 (22149)
Regional	37 (74514)	6 (200)	12 (471)	34 (13761)
Remote or very Remote	23 (45286)	2 (72)	5 (191)	13 (5140)
**Private health insurance**				
No	33 (65036)	35 (1133)	45 (1779)	42 (2734)
Yes	67 (134314)	65 (2080)	55 (2163)	58 (3887)

Compared to Australian-born participants, overseas-born individuals tended to live in major cities. Individuals from Southeast Asia and Northeast Asia were younger and more likely than others to have a university degree and more likely than others to report a lower household income. Table 2 shows the acculturation characteristics of overseas-born individuals according to their region of birth. Compared to European migrants, most Asian-born participants had lived in Australia less than 30 years and migrated to Australia after the age of 30.

**Table 2:** Acculturation characteristics of overseas born participants by region of birthRegion of Birth.

	Northeast Asia	Southeast Asia	Europe
	(n = 3213)	(n = 3942)	(n = 41061)
	%(n)	%(n)	%(n)
**Years lived in Australia**			
0–10	14 (402)	11 (380)	8 (2574)
11–20	39 (1140)	22 (775)	5 (1738)
21–30	26 (774)	41 (1467)	11 (3697)
>30	21 (633)	26 (945)	77 (26317)
**Age migrated to Australia**			
0–10	7 (202)	8 (285)	27 (9208)
11–20	6 (189)	9 (312)	17 (5693)
21–30	20 (594)	28 (991)	32 (10969)
>30	67 (1964)	56 (1979)	25 (8456)

### Self-reported CVD by region of birth

The overall prevalence of self-reported previous CVD within the cohort was 19.5% for men and 11.0% for women. Table 3 shows the crude prevalence of previous CVD (i.e. heart disease, stroke or thrombosis) in Australian-born and overseas-born participants. After adjustment for sex, age and socio-economic variables, the prevalence of CVD was significantly lower for all overseas-born immigrant groups than for Australian-born participants. Northeast Asian migrants had the lowest risk of previous CVD events with an adjusted prevalence ratio (APR) of 0.61 (95%CI 0.54–0.68) compared to Australian-born participants.

**Table 3:** Prevalence of self-reported previous cardiovascular diseases in participants in the 45 and Up Study.

	Previous cardiovascular disease% (n)			PR^1^ (95%CI)		
**Region Of Birth**	**All**	**Male**	**Female**	**All**	**Male**	**Female**
Australia	15 (29948)	19 (17769)	11 (12179)	1	1	1
Northeast Asia	8 (294)	10 (150)	6 (114)	0.61 (0.54–0.68)	0.54 (0.47–0.62)	0.69 (0.58–0.82)
Southeast Asia	10 (375)	14 (222)	7 (153)	0.76 (0.69–0.83)	0.79 (0.70–0.89)	0.72 (0.62–0.84)
Europe	16 (6735)	21 (4344)	13 (2391)	0.92 (0.90–0.94)	0.89 (0.87–0.92)	0.90 (0.87–0.94)

^1^ PR, prevalence ratio, adjusted for age, education, income, private health insurance, marital status and location of residence

### CVD risk factors by region of birth

**Fig 1:**
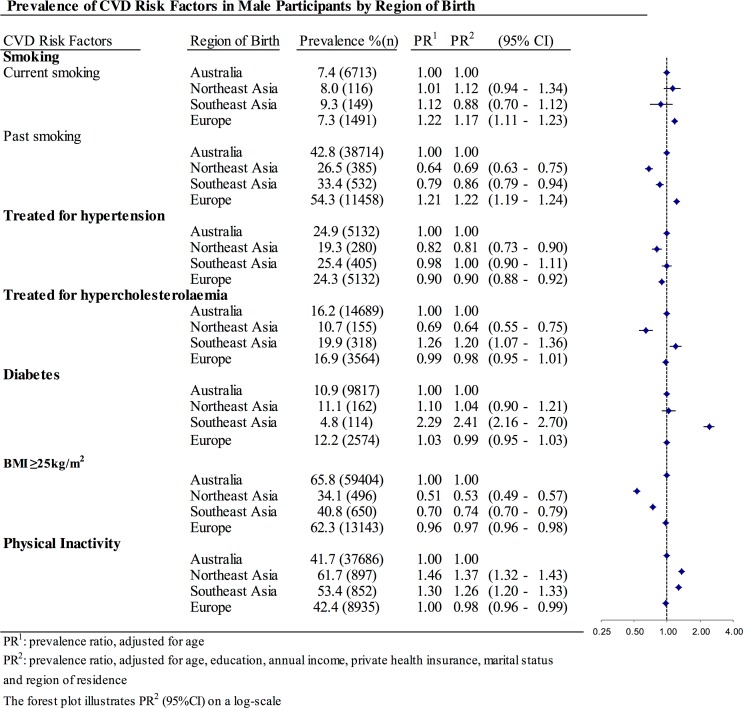
Prevalence of CVD risk factors in male participants according to region of birth.

**Fig 2:**
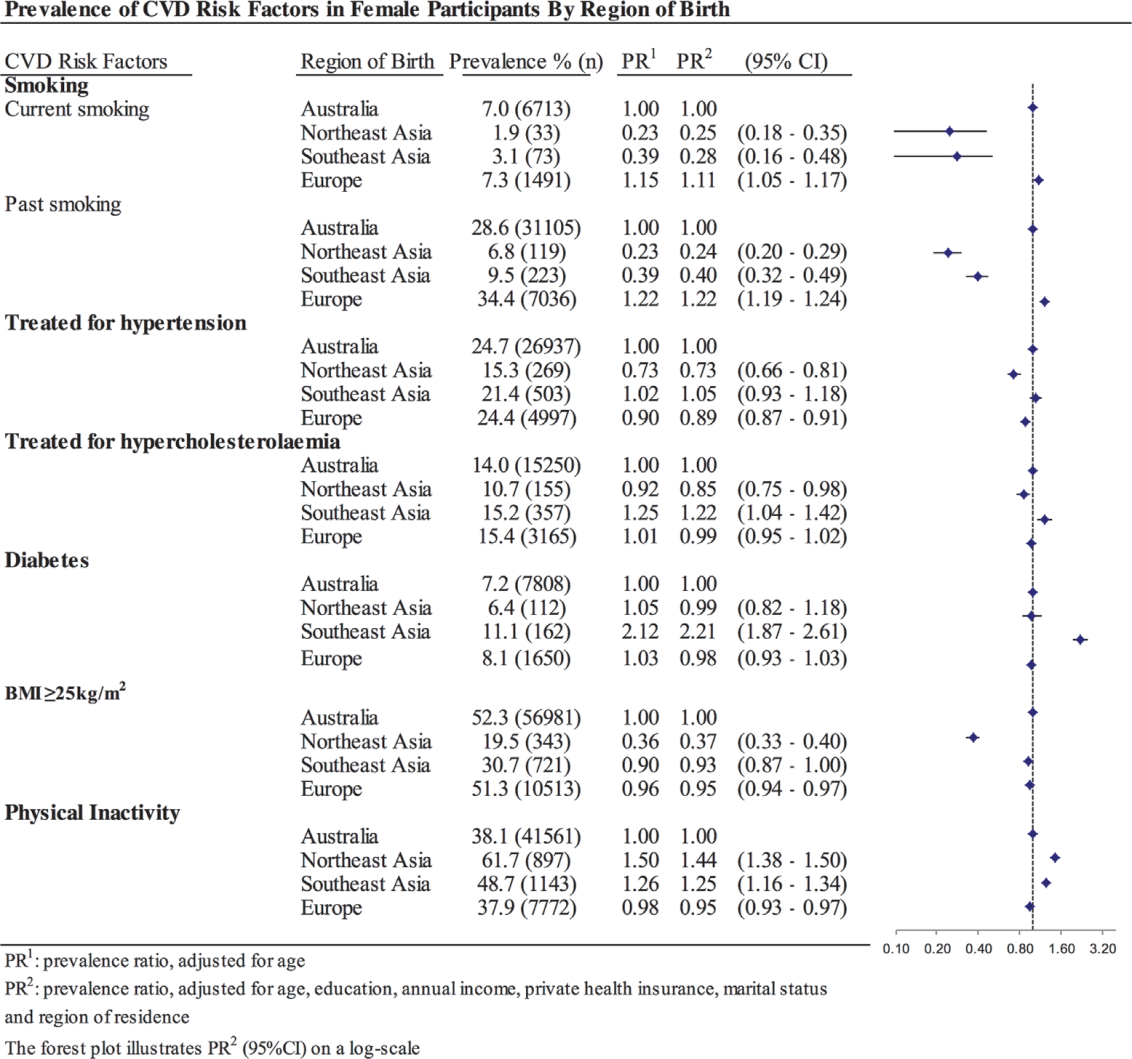
Prevalence of CVD risk factors in female participants according to region of birth.

Asian-born women, but not men, had significantly lower current smoking prevalence than their Australian-born counterparts (Figs. 1 and 2). However, both women and men from Northeast Asia and Southeast Asia were less likely to have smoked in the past. In contrast, European migrants had higher prevalence of both current and past smoking than Australian-born participants.

Northeast Asian migrants were significantly less likely than Australian-born individuals to report current treatment of hypertension and hypercholesterolaemia, but their risk for diabetes was similar (Figs. 1 and 2). Southeast Asian-born participants had generally higher prevalence of all of these three metabolic risk factors compared to Australian-born participants.

**Fig 3:**
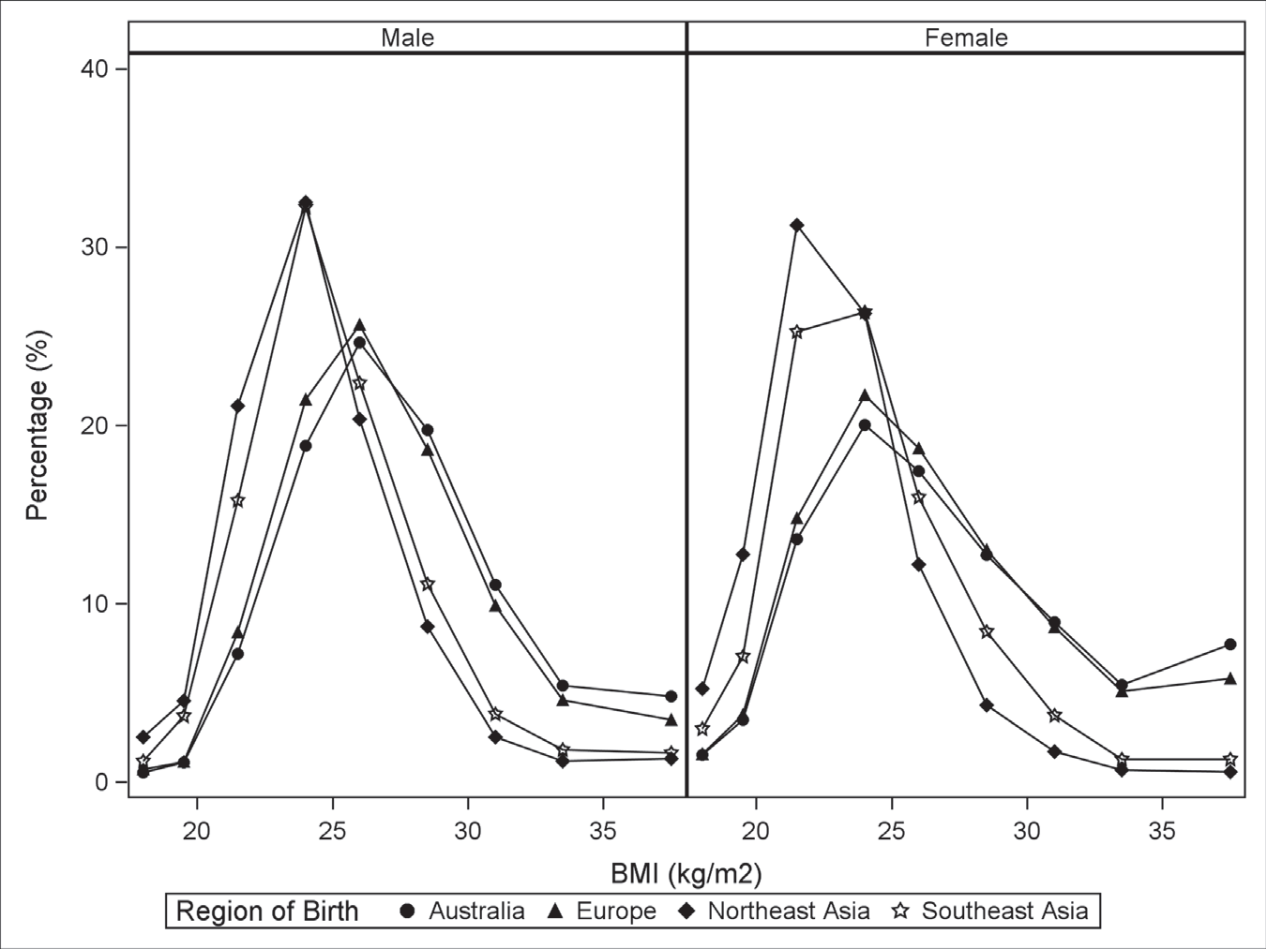
The distribution of body mass index according to region of birth and sex.

Asian-born participants, especially women from Northeast Asia, had a lower average BMI than Australian-born and European-born individuals (Fig. 3). After adjustment for age and socio-economic factors, all of the overseas-born migrant groups were less likely to have a BMI of ≥25kg/m^2^ compared to Australian-born participants. The magnitude of the differences was larger for individuals from Northeast Asia (APR: 0.53 (0.49–0.57) for men and 0.37 (0.33–0.40) for women) than for those from Southeast Asia (APR: 0.63 (0.59–0.67) for men and 0.58 (0.55–0.62) for women) and Europe (APR: 0.97 (0.96–0.98) for men and 0.95 (0.94–0.97) for women).

As studies in Asian populations have shown that a BMI of ≥23kg/m^2^ is associated with increased risk of diabetes, hypertension and other cardiovascular outcomes, we also examined the relationship of cultural background to overweight/obesity defined according to the WHO-recommended BMI cut-off for Asian participants (BMI≥23kg/m^2^). With the different BMI cut-off, men from Southeast Asia had a significantly higher prevalence of overweight/obesity (APR: 1.06 (1.02–1.09)), while women from Southeast Asia were similar to their Australian-born counterparts. Northeast Asian-born participants were still less likely to be overweight, but the magnitude of the difference was smaller. Individuals from both Northeast Asia and Southeast Asia were more likely to be physically inactive than Australian-born and European-born participants.

### CVD risk profiles by region of birth

After adjustment for age and socio-economic factors, compared to Australian-born participants both Northeast Asian and Southeast Asian migrants were more likely to have none of the six major CVD risk factors. However, among those who had at least one risk factor, Northeast Asian-born individuals were more likely to have only a single risk factor, while individuals from Southeast Asia had similar prevalence of multiple risk factors compared with their Australian-born counterparts (Table 4).

**Table 4:** Prevalence of cardiovascular disease risk profiles by region of birth and sex (fully adjusted).

Number of CVD		Male			Female		
Risk Factors	Region of Birth	Prevalence % (n)	PR^1^	(95% CI)	Prevalence % (n)	PR^1^	(95% CI)
**0**							
	Australia	10.6 (9623)	1.00		17.1 (18622)	1.00	
	Northeast Asia	14.7 (213)	1.32	(1.16–1.50)	23.0 (405)	1.25	(1.14–1.36)
	Southeast Asia	13.4 (213)	1.23	(1.08–1.40)	22.6 (530)	1.26	(1.17–1.36)
	Europe	11.6 (2444)	1.12	(1.07–1.16)	18.8 (3859)	1.19	(1.15–1.23)
1							
	Australia	30.3 (27424)	1.00		30.1 (32809)	1.00	
	Northeast Asia	35.5 (516)	1.18	(1.10–1.26)	43.4 (763)	1.41	(1.34–1.49)
	Southeast Asia	29.8 (476)	0.99	(0.92–1.07)	31.4 (737)	1.03	(0.97–1.10)
	Europe	29.8 (6278)	1.03	(1.00–1.05)	34.0 (6137)	1.05	(1.02–1.07)
**2**							
	Australia	27.9 (25244)	1.00		23.9 (26034)	1.00	
	Northeast Asia	27.4 (399)	0.98	(0.90–1.07)	15.9 (279)	0.67	(0.60–0.74)
	Southeast Asia	26.0 (414)	0.92	(0.85–1.00)	21.2 (497)	0.89	(0.82–0.96)
	Europe	28.3 (5962)	1.03	(1.00–1.05)	23.3 (4766)	0.97	(0.95–1.00)
**> = 3**							
	Australia	20.1 (18199)	1.00		15.0 (16385)	1.00	
	Northeast Asia	13.6 (198)	0.66	(0.58–0.75)	8.8 (154)	0.62	(0.53–0.72)
	Southeast Asia	19.8 (315)	0.97	(0.88–1.07)	12.8 (300)	0.89	(0.79–0.99)
	Europe	19.8 (4172)	0.93	(0.90–0.96)	16.0 (3269)	0.96	(0.92–0.99)

^1^ PR, prevalence ratio, adjusted for age, education, income, private health insurance, marital status and location of residence

### Acculturation and CVD risk factors and risk profiles in Asian migrants

**Fig 4:**
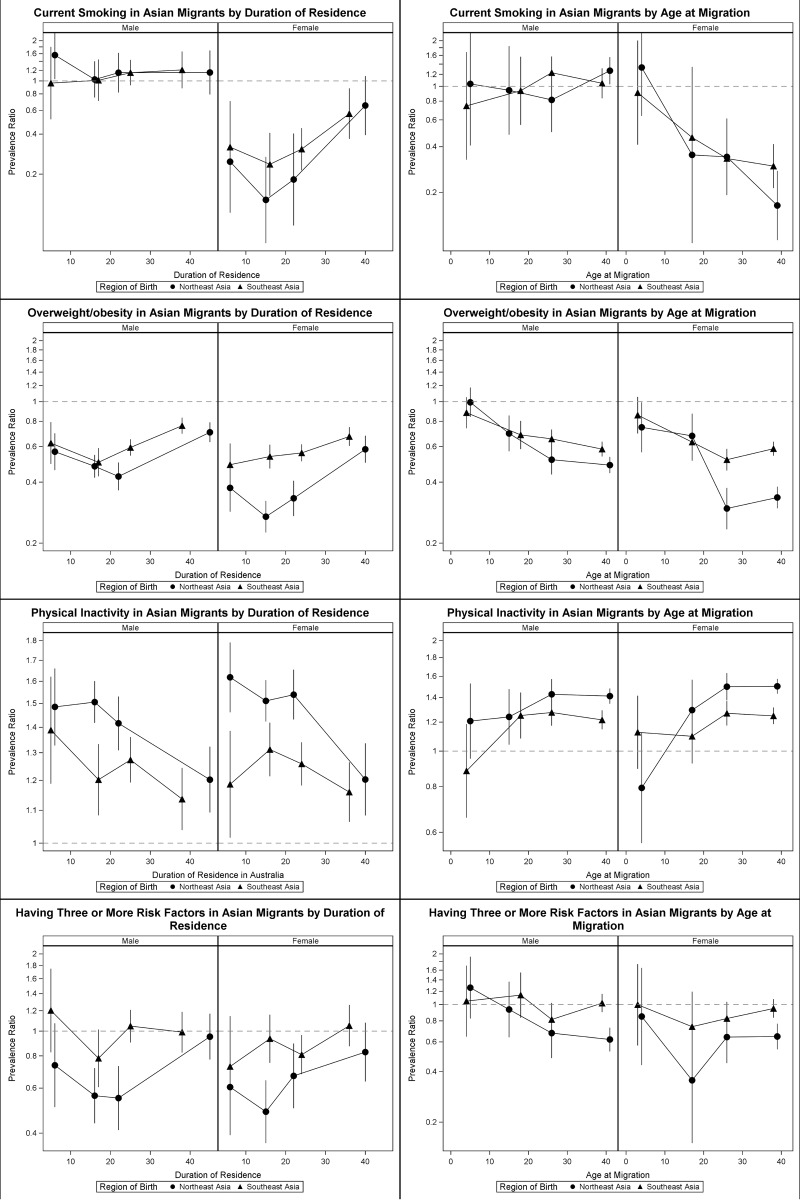
Prevalence ratios of current smoking, overweight/obesity, physical inactivity and having three or more risk factors among men and women migrants from Northeast Asia and Southeast Asia by duration of residence and age of migration compared with Australian-born participants.

Fig. 4 illustrates the prevalence ratios (using Australian-born participants as the reference group) of CVD risk factors according to duration of residence in Australia and age at migration for participants born in Northeast Asia and Southeast Asia. Data are shown only for the four CVD risk factors for which there was a significant trend in prevalence according to the acculturation variables; data for the other variables are presented in S1 Table.

Younger age at migration and increasing duration of residence in Australia were associated with increasing prevalence of current smoking among Northeast Asian-born (P(trend) = 0.05 and 0.03, respectively) and Southeast Asian-born women (P(trend)<0.01 and <0.01, respectively). In women who migrated to Australia before the age of 10, the prevalence of smoking was more than six times higher in Northeast Asian-born and more than twice as high in Southeast Asian-born women, compared to those who migrated after the age of 30. These trends were not observed in men.

The prevalence of overweight/obesity (BMI≥25kg/m^2^) was significantly higher with longer duration of residence in Australia and younger age at migration (e.g. before 10 years old) among Northeast Asian-born and Southeast Asian-born individuals. This trend was not linear for some groups. Younger age at migration and increasing duration of residence were associated with decreasing prevalence of physical inactivity only among Northeast Asian-born men (P(trend) = 0.04 and 0.07, respectively) and women (P(trend)<0.01 and <0.01, respectively).We found no evidence of significant trends in prevalence of diabetes or treatment of hypertension and hypercholesterolaemia in association with the degree of acculturation (S1 Table).

The proportion of people with 3 or more risk factors significantly increased among Northeast Asian-born men (P-trend = 0.04) and Southeast Asian-born women (P-trend = 0.05) with longer duration of residence in Australia.

Results of the sensitivity analysis separating participants into those with and without previous CVD are shown in Table 5. Except for current smoking, the prevalence of each CVD risk factor was higher among participants with previous CVD, for each region of birth and for men and women. However, the association between region of birth and CVD risk factors was similar in participants with and without previous CVD.

**Table 5:** Sensitivity Analysis of Region of Birth and CVD Risk Factors in participants with and without self-reported previous CV.

		Without previous CVD						With previous CVD					
		Male			Female			Male			Female		
CVD Risk Factors	Region of Birth	Prevalence % (n)	PR^1^	(95% CI)	Prevalence % (n)	PR^1^	(95% CI)	Prevalence % (n)	PR^1^	(95% CI)	% (n)	PR^1^	(95% CI)
**Smoking**													
Current smoking	Australia	7.9 (5751)	1.00		7.1 (6897)	1.00		5.4 (962)	1.00		5.7 (692)	1.00	
	Northeast Asia	8.3 (108)	1.10	(0.92–1.33)	1.8 (30)	0.24	(0.17–0.34)	5.3 (8)	1.21	(0.64–2.30)	2.6 (3)	0.57	(0.19–1.72)
	Southeast Asia	10.1 (139)	1.10	(0.94–1.29)	3.3 (72)	0.37	(0.30–0.47)	4.5 (10)	0.68	(0.37–2.30)	0.7 (1)	0.57	(0.15–2.13)
	Europe	8.2 (1380)	1.13	(1.07–1.20)	7.5 (1350)	1.11	(1.05–1.17)	6.6 (291)	1.37	(1.21–1.55)	5.8 (141)	1.18	(1.00–1.41)
Past smoking	Australia	40.4 (29365)	1.00		28.5 (27584)	1.00		52.6 (9349)	1.00		28.9 (3521)	1.00	
	Northeast Asia	24.9 (324)	0.69	(0.62–0.75)	6.6 (109)	0.24	(0.20–0.29)	40.7 (61)	0.81	(0.67–0.99)	8.7 (10)	0.32	(0.18–0.58)
	Southeast Asia	30.9 (424)	0.84	(0.78–0.91)	9.3 (205)	0.33	(0.29–0.38)	48.7 (108)	1.00	(0.88–1.15)	11.8 (18)	0.40	(0.26–0.61)
	Europe	51.9 (8660)	1.23	(1.21–1.25)	34.0 (6137)	1.21	(1.18–1.24)	63.5 (2798)	1.20	(1.17–1.23)	37.2 (902)	1.30	(1.23–1.38)
**Treated for hypertension**													
	Australia	21.8 (15864)	1.00		22.3 (21583)	1.00		37.6 (6680)	1.00		44.0 (5354)	1.00	
	Northeast Asia	17.3 (226)	0.83	(0.74–0.96)	13.9 (228)	0.73	(0.65–0.82)	36.0 (54)	0.94	(0.76–1.17)	36.0 (41)	0.83	(0.65–1.06)
	Southeast Asia	22.9 (315)	1.12	(1.02–1.24)	19.8 (435)	0.88	(0.86–0.91)	40.5 (90)	1.08	(0.92–1.27)	44.4 (68)	1.09	(0.91–1.30)
	Europe	21.5 (3584)	0.90	(0.87–0.93)	21.8 (3943)	1.04	(0.96–1.13)	35.1 (1548)	0.93	(0.89–0.97)	43.5 (1054)	0.96	(0.91–1.01)
**Treated for hypercholesterolaemia**													
	Australia	13.0 (9476)	1.00		12.2 (11790)	1.00		29.3 (5214)	1.00		28.4 (3460)	1.00	
	Northeast Asia	9.2 (120)	0.69	(0.58–0.82)	9.7 (159)	0.88	(0.76–1.02)	23.3 (35)	0.78	(0.58–1.04)	25.4 (29)	0.87	(0.64–1.19)
	Southeast Asia	17.6 (242)	1.34	(1.19–1.51)	14.3 (313)	1.30	(1.17–1.44)	34.2 (76)	1.10	(0.91–1.32)	28.8 (44)	1.03	(0.81–1.32)
	Europe	13.6 (2268)	0.99	(0.95–1.03)	13.6 (2248)	0.99	(0.95–1.03)	29.4 (1296)	1.02	(0.97–1.07)	29.6 (717)	1.01	(0.95–1.08)
**Diabetes**													
	Australia	8.9 (6481)	1.00		6.0 (5384)	1.00		18.8 (3336)	1.00		16.2 (1974)	1.00	
	North East Asia	9.9 (129)	1.11	(0.94–1.31)	11.1 (162)	1.05	(0.86–1.27)	22.0 (33)	1.14	(0.85–1.55)	14.9 (17)	0.93	(0.60–1.44)
	South East Asia	12.7 (174)	1.44	(1.25–1.66)	10.1 (221)	1.75	(1.54–1.99)	28.4 (63)	1.50	(1.21–1.86)	25.5 (39)	1.62	(1.22–2.14)
	Europe	10.1 (1692)	0.99	(0.94–1.04)	6.9 (0.55)	0.99	(0.93–1.05)	20.0 (882)	1.05	(0.98–1.12)	17.1 (413)	1.00	(0.91–1.10)
**BMI≥25kg/m2**													
	Australia	65.9 (47866)	1.00		52.0 (50308)	1.00		64.9 (11538)	1.00		54.8 (6673)	1.00	
	Northeast Asia	33.7 (440)	0.52	(0.48–0.56)	19.0 (313)	0.36	(0.32–0.40)	37.3 (56)	0.63	(0.51–0.77)	26.3 (30)	0.48	(0.36–0.57)
	Southeast Asia	40.9 (561)	0.63	(0.59–0.67)	30.3 (650)	0.58	(0.54–0.62)	61.8 (89)	0.63	(0.54–0.74)	36.0 (55)	0.64	(0.52–0.79)
	Europe	62.5 (10431)	0.97	(0.96–0.98)	50.8 (9173)	0.95	(0.93–0.96)	61.6 (2712)	0.98	(0.96–1.01)	55.3 (1340)	1.01	(0.97–1.05)
**Physical Inactivity**													
	Australia	40.4 (29340)	1.00		37.2 (37686)	1.00		47.0 (8346)	1.00		47.5 (5537)	1.00	
	Northeast Asia	61.2 (798)	1.40	(1.34–1.47)	57.7 (949)	1.46	(1.40–1.52)	66.0 (99)	1.27	(1.13–1.42)	61.4 (70)	1.29	(1.12–1.48)
	Southeast Asia	53.8 (738)	1.25	(1.19–1.31)	47.9 (1050)	1.23	(1.17–1.28)	51.4 (114)	1.03	(0.91–1.17)	60.8 (93)	1.29	(1.14–1.45)
	Europe	41.2 (6869)	0.98	(0.96–1.00)	36.8 (6643)	0.95	(0.93–0.97)	46.9 (2066)	0.97	(0.93–1.00)	46.6 (1129)	0.95	(0.91–1.00)

^1^ PR, prevalence ratio, adjusted for age, education, income, private health insurance, marital status and location of residence

## Discussion

We have found that the prevalence of self-reported CVD within the 45 and Up Study cohort was significantly lower for all overseas-born immigrant groups than for Australian-born individuals. However, there was significant variation in the risk factor profiles and the proportion of at-risk individuals for each CVD risk factor across different migrant subgroups. Northeast Asian-born participants had significantly more favourable CVD risk profiles. In spite of the lower prevalence of overweight/obesity, Southeast Asian-born participants had a higher prevalence of certain metabolic risk factors, compared with Australian-born participants.

Consistent with our findings, a much lower prevalence of current smoking among Asian-born women in comparison to Asian-born men and individuals of host countries has been reported in studies from the United States [25–27], Canada [28] and Australia[12]. One possible explanation is that there are different social norms regarding gender and smoking in Asian and western countries. Smoking is commonly viewed as a masculine behaviour in many Asian countries, and as not acceptable for women [25,29]. However, despite impressive tobacco control measures in Australia, we found that the prevalence of smoking increased in association with longer duration of residence in Asian-born women from the 45 and Up Study, probably because of changes in social norms around smoking as migrants adopt the host culture.

Our finding of a much lower likelihood of overweight/obesity in Asian-born individuals from the 45 and Up Study is consistent with results from other studies using the same BMI cut-off (25 kg/m^2^ or 30 kg/m^2^) for Asian migrants and individuals of host countries[14,30]. However when a recommended BMI cut-off for Asians of ≥23 was used, a significantly higher proportion of Southeast Asia-born men and a similar proportion of Southeast Asia-born women were overweight or obese compared to their Australian-born counterparts. Northeast Asian-born participants were still less likely to be overweight/obese.

Despite the lower or similar BMI levels in Southeast Asian- versus Australian-born individuals from the 45 and Up Study, Southeast Asian migrants had a higher prevalence of diabetes, hypertension and hypercholesterolemia [15,31,32]. This is consistent with existing evidence which indicates that the prevalence of diabetes and other cardio-metabolic risk factors and visceral adiposity are greater at a lower mean BMI in Asian populations compared to people of European descent [33–36].

Our data also show that migration to Australia at a younger age, and a longer duration of residence in Australia were associated with an increased prevalence of current smoking and overweight/obesity and with increased physical activity in some Asian migrant subgroups within the 45 and Up Study cohort.

The age at migration, and the duration of residence in a new country, are both considered to be indirect proxies for acculturation. Previous studies have shown that the prevalence of smoking and overweight/obesity in Asian-born individuals converges toward that of the population born in the host country in association with longer duration of residence and younger age at migration [12,25,37,38]. Despite the negative effect of acculturation on many CVD risk factors, our findings are consistent with others from North America that show a trend toward higher levels of physical activity with greater acculturation in ethnically diverse groups of migrants [39,40].

We found that longer duration of residence in Australia was associated with an increased probability of having three or more risk factors in most of the Asian migrant subgroups within the 45 and Up Study cohort. These findings are consistent with those of Chui et al. in their study of Chinese migrants to Canada [28]. The results suggest that the overall health advantage of being a migrant becomes less evident, in Northeast Asian-born individuals, as they live longer in a western country.

Our findings have implications for the planning of health promotion strategies in migrant populations. Efforts should be made to retain normal BMIs in both men and women and the low prevalence of smoking in women, as migrants become acculturated to the host country. At the same time, migrants could be encourage to adopt the healthy aspects of the lifestyle of the host country, such as increasing physical activity.

A range of cut-points have been used in past studies to summarize time since migration, with “long term” residence of >10 years or >15 years commonly used to define acculturation. Our study focused on a cohort aged 45 years and over, having a relatively high prevalence of CVD risk factors, which is distinct from previous studies and allowed us to examine the relationship of incremental changes in the acculturation variables and CVD risk factors over a longer time frame (e.g. lived in Australia for more than 30 years). We are thus able to provide greater detail on the gradients in risk in older “long term” residents in Australia, showing us that there is a significant change in some CVD risk factors over this longer time period. Previous studies have suggested that many changes in health behaviours in migrant groups take decades to appear [41].

There are some limitations to this analysis. The 45 and Up Study is a cohort study, with an estimated response rate of 17.9%; hence, the absolute prevalence of CVD risk factors may not directly reflect that of the general population. However, relative risks from internal comparisons within the cohort, such as those reported here, have been shown to be generalizable[42]. Most of the data are self-reported. There may be a higher proportion of undiagnosed and untreated metabolic risk factors, such as impaired glucose tolerance and hypertension, in disadvantaged migrants who are less likely to seek health care. However, validation studies involving 45 and Up Study participants have found good agreement between self-reported and measured BMI[43] and shown that self-reported diagnosis of diabetes has high sensitivity and specificity compared to available administrative data collections [44]. The 45 and Up Study questionnaire was available only in English. Non-English speaking migrants with limited English skills were thus less likely to be involved in this study. Reduced participation of less acculturated and more disadvantaged migrants may lead to more conservative estimates for differences in the prevalence of CVD risk factors [45]. Furthermore, these results were derived from cross-sectional data; we were therefore unable to draw conclusions about the causal relationship between acculturation level and increased cardiovascular risk. The results may be influenced by the cohort effect, such that different waves of migrants may have had different cardiovascular risk profiles prior to immigration.

## Conclusion

In this study, we found contrasting CVD risk profiles in migrants from Northeast Asia and Southeast Asia. Northeast Asian migrants had a generally favourable CVD risk profile while Southeast Asian-born participants were equally as likely as Australian-born individuals to have multiple risk factors and to have a higher prevalence of certain metabolic risk factors. With greater acculturation, general increases in CVD risk factors were seen among both Northeast and Southeast Asian migrants, including increases in the prevalence of smoking in women. However, changes in patterns of physical activity with acculturation were generally favourable. Given the substantial increase in migration from Asia to Australia in recent decades, these findings highlight the potential importance of developing health promotion strategies to preserve healthy lifestyles and address specific risk factors, in Asian communities.

## Supporting Information

S1 Table Cardiovascular risk factors for all Asian-born participants by acculturation status. (XLSX)
